# F﻿irst detection ﻿of herpesvirus and hemosporidians in the endangered Pyrenean Capercaillie (*Tetrao urogallus aquitanicus*)

**DOI:** 10.1038/s41598-023-48123-3

**Published:** 2023-12-11

**Authors:** Olga Nicolás de Francisco, Irene Sacristán, Ana Carolina Ewbank, Roser Velarde, Ivan Afonso, Diego Garcia-Ferré, Bárbara Martín-Maldonado, Fernando Esperón, Irene Iglesias, Ana de la Torre, Antoni Margalida, Carlos Sacristán

**Affiliations:** 1 Department of Forest Management and Natural Environment, School of Veterinary Medicine, University of Lleida, Lleida, Spain; 2 Group of Epidemiology and Environmental Health, Animal Health Research Centre (INIA-CISA), Valdeolmos, Spain; 3 Laboratory of Wildlife Comparative Pathology–LAPCOM, School of Veterinary Medicine and Animal Sciences, University of São Paulo, São Paulo, Brazil; 4 Wildlife Ecology & Health Group (WE&H) and Servei d’Ecopatologia de Fauna Salvatge (SEFaS), Departament de Medicina i Cirurgia Animal, Universitat Autònoma de Barcelona, Bellaterra, Spain; 5 Natural Environment Department, Conselh Generau d’Aran, Vielha, Spain; 6 Flora and Fauna Service, Department of Climatic Action, Food and Rural Agenda (Government of Catalonia), Barcelona, Spain; 7 Department of Veterinary, Faculty of Biomedical and Health Sciences, Universidad Europea de Madrid, Villaviciosa de Odón, Spain; 8 Pyrenean Institute of Ecology (CSIC), Jaca, Spain

**Keywords:** Ecology, Diseases

## Abstract

Pathogens affect wild bird populations worldwide, contributing to their decline. Considering the scarce health data regarding the endangered Pyrenean Capercaillie (*Tetrao urogallus aquitanicus*), we molecularly surveyed selected pathogens (Newcastle disease virus, Avian influenza virus, *Chlamydia psittaci*, avian pathogenic *Escherichia coli*, *Campylobacter jejuni*, and *Salmonella* spp.) in 30 Pyrenean Capercaillie feces collected in the field (Catalonia, northeastern Spain). Additionally, swab and tissue samples from eight wild Pyrenean Capercaillies of Catalonia and Andorra were molecularly tested for herpesvirus and hemosporidians (*Plasmodium* spp., *Haemoproteus* spp., and *Leucocytozoon* spp.). All fecal samples were negative for the pathogens tested. Nevertheless, we detected a novel herpesvirus in 50% (4/8) of the Pyrenean Capercaillies, and hemosporidian DNA in 62.5% (5/8) of the tissue samples (i.e., *Haemoproteus* sp. [4 of 8] and/or *Leucocytozoon* sp. [3 of 8]). To our knowledge, this is the first detection of herpesvirus and hemosporidians infections in Pyrenean Capercaillies. The putative novel herpesvirus belongs to the genus *Iltovirus*. The presence of hemosporidian parasites in this mountain bird species is of concern, and could be related to the marked increase in the average temperature in the Pyrenees as a consequence of climate change. Our findings are fundamental to improve the conservation plans for the endangered Pyrenean Capercaillie population.

## Introduction

The Pyrenean Capercaillie (*Tetrao urogallus aquitanicus*) is a very elusive endangered subspecies of Western Capercaillie (*T. urogallus*, order Galliformes) that sustains local endemism in the Pyrenees and inhabits narrow elevation ranges^1^. This mountain bird is highly sensitive to environmental changes and stress^2^. Several factors are considered threats to Pyrenean Capercaillies, including climate change, habitat fragmentation, increased extensive livestock and wild ungulate populations, and disturbances resulting from off-track practices^2^. Additionally, increased temperatures may promote their exposure to novel pathogens that were not originally present and/or that normally belong to other altitudinal ranges^3^. Responses to these environmental stressors may lead to increased stress hormone levels, immunosuppression and increased susceptibility to environmental changes and/or diseases^4,5^.

Pathogens may contribute to wild bird population decline due to mortality and decreased reproductive success^6,7^. Furthermore, some wild bird infectious agents have zoonotic potential and/or relevance in poultry^7,8^. To this date, there are no reports of pathogens affecting Pyrenean capercaillie, and their descriptions in other Wester Capercaillie subspecies are very scarce, limited to infections by Usutu virus, *Plasmodium relictum*, *Capillaria* sp., and *Eimeria* spp. in a Western Capercaillie (*T. u. crassirostris*) kept in captivity in Switzerland^9^, *Haemoproteus* sp. in two Western Capercaillies from Austria^10^, and *Escherichia coli*, *Clostridium perfringens*, *Enterococcus* spp. or *Aspergillus fumigatus* in Cantabrian Capercaillie (*T. u. cantabricus*) from a breeding center in Spain^11^.

The aim of this study was to generate basic health data and investigate potential factors contributing to the current decline of the Pyrenean Capercaillie population. Thus, we molecularly surveyed the presence of Avian influenza virus, Newcastle virus, *Chlamydia psittaci*, avian pathogenic *E. coli (*APEC), *Campylobacter jejuni* and *Salmonella* spp. in Pyrenean Capercaillie fecal samples, and tested swab and tissue samples for herpesviruses and hemosporidians (*Plasmodium*, *Haemoproteus* and *Leucocytozoon*).

## Materials and methods

### Study species

The Pyrenean Capercaillie is a subalpine bird, distributed year-round along the Pyrenean range, southwestern Europe^1^. This subspecies was recently reclassified as “Endangered” by the Spanish List of Wildlife Species under Special Protection Regime^12^.

### Study area

**Figure 1:**
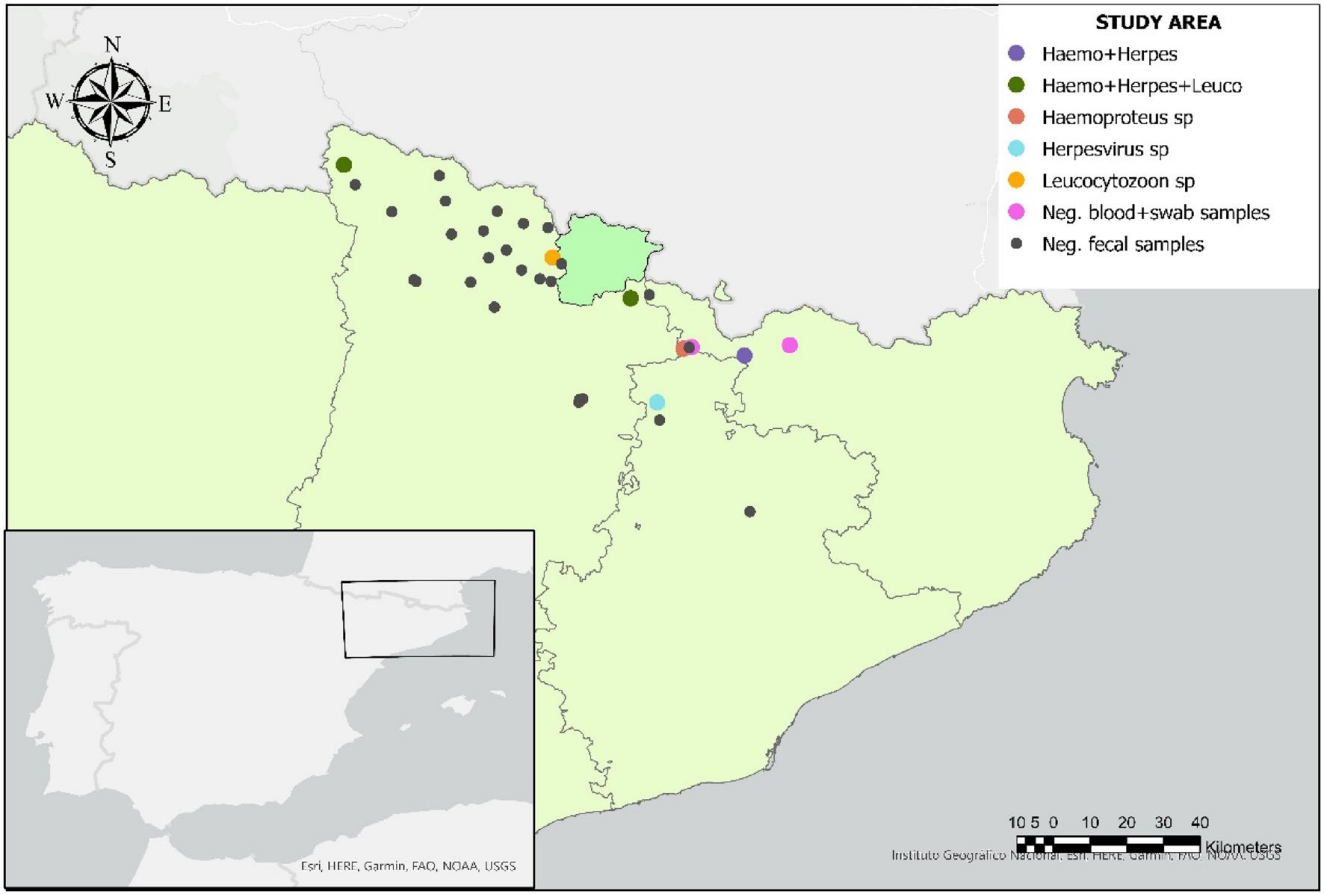
Location of the Pyrenean Capercaillies (*Tetrao urogallus aquitanicus*) sampled for the study and results of the herpesvirus and *Leucocytozoon* sp. and *Haemoproteus* sp. analyses. The figure was created with ArcGIS Pro v3.1 (https://www.esri.es/es-es/arcgis/productos/arcgis-pro/introduccion).

The study was performed in the Catalonian Pyrenees (Val d’Aran, Pallars Sobirà, Pallars Jussà, Alt Urgell, Cerdanya, Berguedà, and Ripollès counties, Spain), and Andorra (Fig. 1), from 1571 to 2302 m above sea level (a.s.l.). These territories are placed in the axial Pyrenees (Val d’Aran, Pallars Sobirà, Cerdanya, Andorra), Pre-Pyrenees (Pallars Jussà, Berguedà) or in the Pre-Pyrenees, with some areas in the Pyrenees (Alt Urgell, Ripollès).

### Samples

#### Fresh fecal samples

Fresh fecal samples (n = 30) were collected in the field during the spring, summer and fall of 2020 and 2021, from areas within the species’ distribution range in the Catalonian Pyrenees (Val d’Aran [n = 3], Pallars Sobirà [n = 13], Pallars Jussà [n = 1], Alt Urgell [n = 7], Cerdanya [n = 1], Berguedà [n = 2], and Ripollès [n = 3] counties). The characteristic morphological features of capercaillie feces facilitated their identification (Supplementary Fig. 1). Fecal samples were collected with latex gloves and sterilized lingual depressors, stored without media in autoclaved microtubes, and kept frozen at − 20/− 80 °C until processed.

#### Tissue and swab samples

We collected tissue samples (blood, central nervous system, lung, liver, spleen, and kidney) from eight adult male Pyrenean Capercaillies (Table 1). Six of these animals were sampled (blood, oropharyngeal and/or cloacal swab samples) during Pyrenean Capercaillie spring captures for tagging in Catalonia (Spain), and two additional cases were found in the field in Catalonia and Andorra (Fig. 1, Table 1). Specifically, blood samples (5 to 7 ml) from five individuals were taken from the brachial vein using 10 ml syringes (23-gauge, 25 mm needles), during sedation procedures for GPS placement. Blood samples were collected in EDTA and biochemistry tubes, kept refrigerated, and used to perform a complete hematology and biochemical study (data not shown), while a volume of 0.5 ml of whole blood was kept frozen (− 20 ºC) for molecular studies. Oropharyngeal (n = 5) and cloacal (n = 5) swab samples were taken during captures. Additionally, tissue samples aside from blood (i.e., central nervous system, lung, liver, kidney, spleen, and kidney) were taken during the necropsy of five individuals (22038–22040, 22163, 23001, Table 1). Necropsies were conducted following a standard protocol^13^.

**Table 1:** Individual identification (ID#), county of origin, analyzed tissues, year of capture/death, and results of Apicomplexa PCR (*Haemoproteus* spp. [*Haem*], *Plasmodium* spp. [*Plasm*.] and *Leucocytozoon* spp. [*Leucocy*.]) and herpesvirus PCR of the adult male Pyrenean Capercaillies (*Tetrao urogallus aquitanicus*) analyzed in this study.

ID#	County of origin	Height at sampling point (m)	Year of capture/death and additional information	Analyzed tissues	Apicomplexa PCR result			Herpesvirus PCR result
					*Haem*	*Plasm*	*Leucocy*	
22036	Cerdanya	1873.9	Captured in 2022	Blood	+	−	−	−
				Oropharyngeal swab	NT	NT	NT	NT
				Cloacal swab	NT	NT	NT	NT
22037	Berguedà	1953.4	Captured in 2022	Blood	−	−	−	−
				Oropharyngeal swab	NT	NT	NT	−
				Cloacal swab	NT	NT	NT	+
22038	Ripollès	1654.5	Died after capture and sedation for GPS-placement in 2022, during recovery	Blood	−	−	−	−
				Brain	+	−	−	+
				Lung	+	−	−	−
				Liver	+	−	−	−
				Kidney	+	−	−	−
				Oropharyngeal swab	NT	NT	NT	−
				Cloacal swab	NT	NT	NT	+
22039	Cerdanya	2255.3	Died after capture, during sedation for GPS-placement, in 2022	Brain	−	−	−	−
				Lung	+	−	+	−
				Liver	+	−	+	−
				Kidney	+	−	−	−
				Oropharyngeal swab	NT	NT	NT	−
				Cloacal swab	NT	NT	NT	+
22040	Andorra	2302.0	Dead after trauma (collision against a parked van) in September 2021	Brain	−	−	−	−
				Lung	−	−	−	−
				Liver	−	−	+	−
				Kidney	−	−	−	−
22041	Masella, Cerdanya	2062.5	Captured in 2020	Blood	−	−	−	−
				Oropharyngeal swab	NT	NT	NT	−
				Cloacal swab	NT	NT	NT	−
22163	Vall d’ Aran	1571.7	Found weak and cachectic by excursionist (Oct 20, 2020). Found dead on Oct 23, 2020	Brain	−	−	−	−
				Lung	+	−	+	-
				Liver	+	−	−	+
				Kidney	+	−	−	+
23001	Ripollès	1826.8	Captured, sedated and tagged with GPS in May 6, 2021; found dead in Oct 6, 2022	Blood	−	−	−	−
				Lung	−	−	−	−
				Liver	−	−	−	−
				Spleen	−	−	−	−

*NT* not tested.

### Histopathology

Selected tissue samples were placed in 4% neutral buffered formalin, routinely processed, sectioned at 3–4 μm, stained with hematoxylin and eosin, and examined under light microscopy. Sections included brain, trachea, thyroid and parathyroid glands, lungs, heart, liver, kidney, spleen, skeletal muscle, adrenal gland, pancreas, testis, esophageal-proventricular junction, and any gross lesion detected during necropsy. In all cases, intestinal samples were in advanced stages of autolysis, which prevented histopathological examination.

### Blood smear

Blood smears were performed immediately after sampling and stained with May-Grunwald-Giemsa. In cases where postmortem samples were collected, blood was sampled from the heart. Three blood smears were performed in each of these animals, and stained with a wright stain (Sigma-Aldrich Inc., St. Louis, USA) in an automatic stainer Hematek 2000 (Siemens Healthcare Diagnostic Inc., Tarrrytown, NY, USA),

### Molecular methods

#### Fecal samples

Fecal samples (n = 30) were thawed at room temperature, and aliquots of 0.2 mg were processed for simultaneous RNA and DNA extraction by pressure filtration (QuickGene DNA tissue kit S, Fujifilm), following the manufacturer’s instructions, and added RNA carrier supplementation during lysis. Real-time PCR (rt-PCR) protocols were used to detect *Salmonella* spp. and *C. jejuni*^14^. *E. coli* was tested by a conventional PCR followed by an octuplex PCR for avian pathogenicity factors^15^. Avian influenza virus and Newcastle virus RNA, and *Ch. psittaci* DNA were detected using rt-PCR protocols, respectively based on TaqMan probe^16,17^, and on high resolution melting (HRM) analysis^18^.

#### Blood and tissue samples

Solid tissue (n = 19) samples were mechanically homogenized using 20 mg of solid tissue with 300 µl PBS, 200 µl lysis buffer and ceramic beads placed in 2 ml tubes. Cloacal (n = 5) and oropharyngeal (n = 5) swab samples were mixed with 300 µl of PBS and 200 µl of lysis buffer, vortexed for 1 min, mixed with 25 µl proteinase K and 5.9 µl of RNA carrier, and vortexed for 1 min. Blood samples (n = 5, 200 µl each) were directly extracted. Total DNA and RNA from these samples were extracted using the PureLink Viral RNA/DNA Mini Kit (Invitrogen, Waltham, Massachusetts, USA), following the manufacturer’s instructions. The extractions of blood, solid tissues, oropharyngeal and/or cloacal samples of eight animals were tested against herpesviruses using a nested broad-spectrum PCR that amplifies a fragment (215–315 bp) of DNA polymerase (DPOL) gene^19^. The extracted samples (excluding swabs) from the eight animals were also analyzed for *Plasmodium* spp., *Haemoproteus* spp., and *Leucocytozoon* spp. with a nested PCR that partially amplifies the mitochondrial cytochrome b gene of Apicomplexan hemoparasites^20^. The internal PCR has a specific pair of primers to amplify an approximately 480 bp fragment of *Plasmodium* spp. and *Haemoproteus* spp., and another one for *Leucocytozoon* spp. (also around 480 bp)^20^.

Amplicons of the expected size were purified with Exo-SAP IT (Thermo Fisher Scientific) and directly sequenced in both directions. The consensus sequences were constructed using the ClustalW alignment of forward and reverse sequences in MEGA 7.0^21^ and Geneious^®^. The obtained consensus sequences, without primers, were compared to the closest ones from GenBank/EMBL/DDBJ by BLAST search, and the percentage of nucleotide and amino acid (aa) similarity was calculated based on p-distance using MEGA 7.0 software, as described by Ewbank et al.^22^. Herpesvirus and hemosporidian maximum likelihood phylograms were constructed with MEGA 7.0 and RaXM 1.5^23^, data set was resampled 1000 times for bootstrap values, and the evolutionary model was selected with ProtTest (v3.4.2). To construct the hemosporidians phylograms, previously identified representative lineages were obtained from MalAvi database.

### Ethics approval

The Pyrenean Capercaillie captures included in this article were carried out on public lands, in strict compliance with the European (Directives 92/43/CEE and 147/2009/CE), Spanish (Act 42/2007 and Act 12/1985), and Catalan (Decree 148/1992, of 9 June) regulations regarding photographic, scientific, and sports activities that may affect wildlife species and Legislative Decree 2/2008, of April 15, approving the Revised Text of the Animal Protection Act). Exceptional permits for trapping, sedation, sampling, movement, and equipping the target species with transmitters -protected under the Spanish law- were obtained from the competent authorities (Capercaillie capture and sedation permits number: SF/005/2020, SF/002/2020, SF/036/2020, SF/0089/2021, SF/0029/2022–Department of Environment and Housing and Alt Pirineu Natural Park; Government of Catalonia). The protocols for trapping, movement, sedation, tagging and sampling the Pyrenean Capercaillies were consistent with the best practices and technical and scientific recommendations related to animal welfare of European (Directives 92/43/CEE and 147/2009/CE), Spanish (Act 42/2007 and Act 12/1985), and Catalan (Decree 148/1992, of 9 June) regulations and were approved by Government of Catalonia. Ethic committee code for Pyrenean Capercaillies captures and sedation was “2/2020/DG”, granted the Climatic Action, Food and Rural Agenda of the Government of Catalonia.

### ARRIVE guidelines

Not applicable, given that these guidelines are for reporting in vivo experiments.

## Results

### Anatomopathological findings

Necropsies were performed in five Pyrenean Capercaillies (cases 22038–22040, 22163, 23001), and histopathology was conducted in four of these birds. The main gross and histopathologic findings are described in Supplementary Table 1. Of note, none of the examined animals presented lesions consistent with those associated with herpesvirus, *Plasmodium*, *Haemoproteus* or *Leucocytozoon* (see below). Specifically, exo-erythrocytic development of haemoproteids (meronts and megalomeronts) were not identified in any of the available tissue sections.

### Blood smear

**Figure 2:**
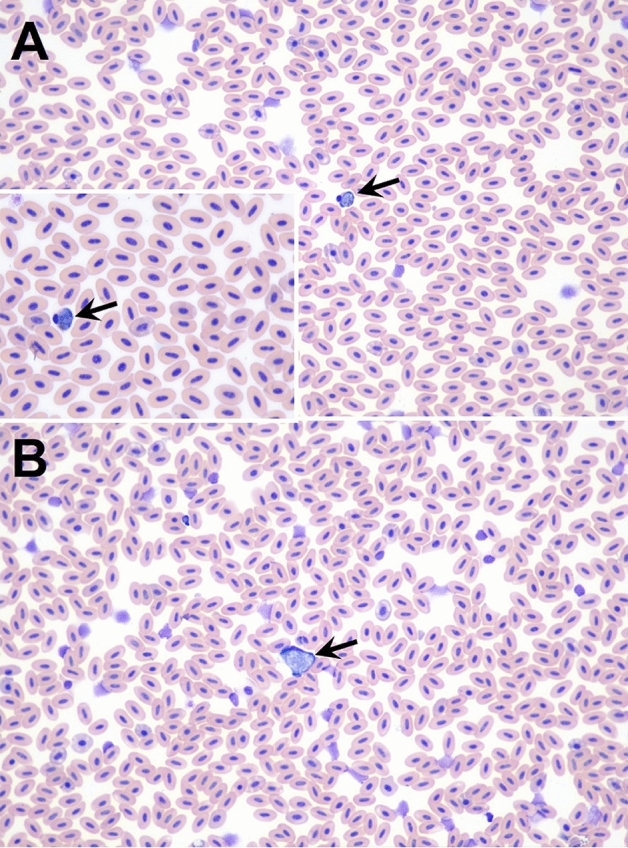
Blood smear from Pyrenean Capercaillie (*Tetrao urogallus aquitanicus*) case number 22039 from Lles de Cerdanya county (Catalonia, Spain) showing hemoparasites consistent with: (**A**) *Haemoproteus* sp., black arrow, and inset (**B**) *Leucocytozoon* sp. black arrow. ×40, Wright stain.

Blood smears were performed in five animals: 22036–22039, and 22041. Structures consistent with hemoparasites were identified under light microscopy in three animals (i.e., *Haemoproteus*-like structures in animals 22036, 22038, 22039, and *Leucocytozoon*-like structures in case 22039) (Fig. 2)*.*

### Molecular techniques

None of the tested fecal samples was PCR-positive to Avian influenza virus, Newcastle disease virus, *Ch. psittaci*, APEC, *Salmonella* spp. or *C. jejuni.*

**Figure 3:**
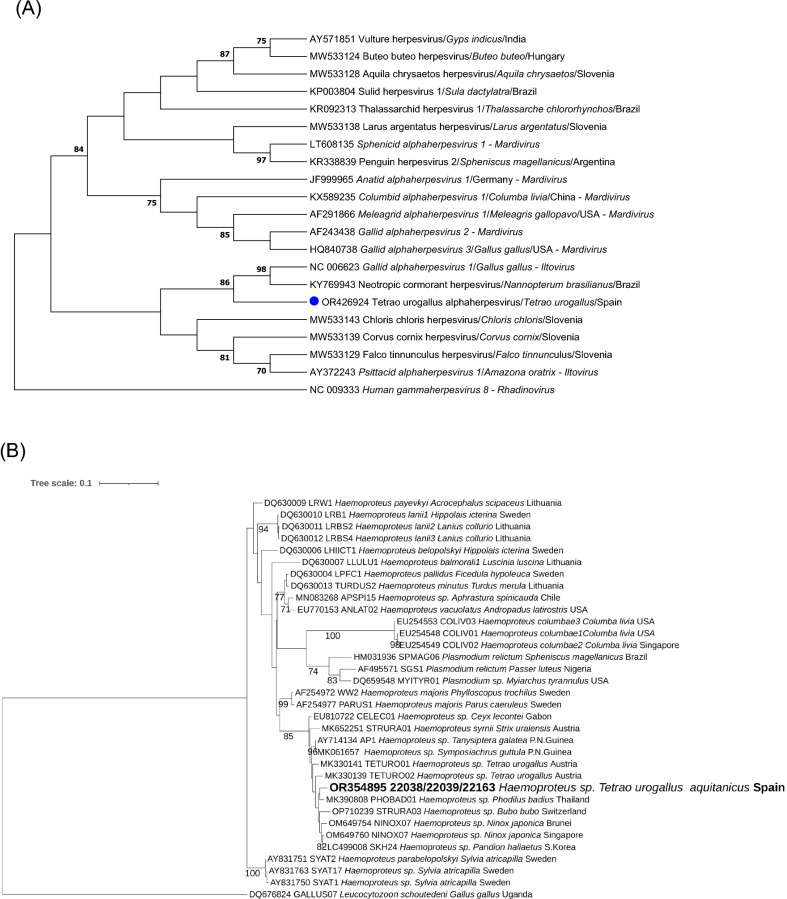

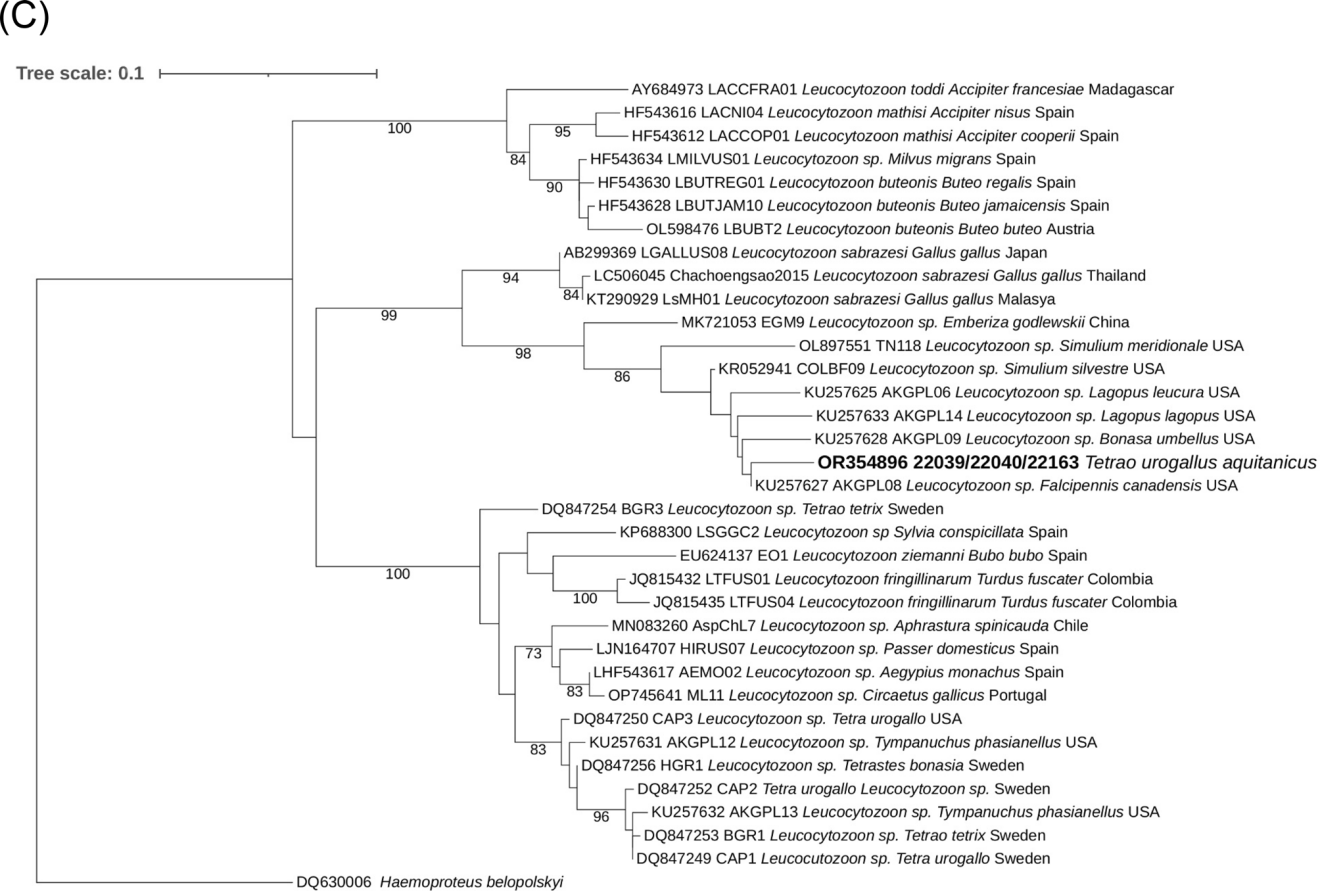
Maximum likelihood phylograms of the ClustalW alignment of: (**A**) The deduced amino acid herpesviral DNA polymerase sequences (i) found in Pyrenean Capercaillies (*Tetrao urogallus aquitanicus*) (blue dot), (ii) the closest sequences from GenBank database, and iii) other alphaherpesvirus sequences described in birds, with *Mardivirus* and *Iltovirus* genera accepted species. *Human gammaherpesvirus 8* was selected as outgroup. The herpesvirus phylogram was based on the gamma distributed Le and Gascuel model with Invariant sites and Gamma distribution (LG + I + G). (**B**) The *Haemoproteus* cytochrome b sequence found in Pyrenean Capercaillies and other birds, with *Leucocytozoon schoutedeni* selected as outgroup; it was based on Gamma distribution (GTRGAMMA). (**C**) The cytochrome b sequence of *Leucocytozoon* sp. found in Pyrenean Capercaillies and other birds, with *Haemoproteus belopolskyi* selected as outgroup, and based on Gamma distribution (GTRGAMMAI). The reliability of all phylograms was tested by a 1000 bootstrap analysis; those bootstrap values lower than 70 were omitted.

Four out of eight animals were herpesvirus-positive (22037, 22038, 22039, 22163) (Table 1). These animals were positive in cloacal swab samples (n = 3), brain (n = 1), liver (n = 1) and kidney (n = 1). The same herpesvirus sequence type was obtained in all sequenced amplicons, and presented the highest nucleotide (79.9%) and aa (80.6%) identities with an alphaherpesvirus sequence retrieved from a Neotropical Cormorant (*Phalacrocorax brasilianus*) of Chile (KY769943) and with several *Gallid alphaherpesvirus 1* sequences from chicken (*Gallus gallus*) (e.g., JX458824, MK895003, MF405079)—the latter also known as infectious laryngotracheitis virus. The obtained sequence clustered in the phylogram (bootstrap value of 86) with a herpesvirus sequence from a Neotropical Cormorant (KY769943) and with a reference sequence of *Gallid alphaherpesvirus 1* (NC_006623) (Fig. 3A).

Overall, Apicomplexa hemoparasites were identified in five out of eight (62.5%) Pyrenean Capercaillies: *Haemoproteus* sp. in four out of eight and *Leucocytozoon* spp. in three out of eight birds (Table 1). Of note, *Haemoproteus*-like structures were present in blood smears of three of the *Haemoproteus*-PCR-positive cases (220036, 22038, 22039), and *Leucocytozoon*-like structures in one of the *Leucocytozoon*-positive cases (22039), coinfected with *Haemoproteus*. None of the samples was PCR-positive to *Plasmodium* spp. The same *Haemoproteus* sequence type was present in all positive capercaillies, and had the highest nucleotide similarity (98.1%) with *Haemoproteus* sp. sequences of birds of Thailand (MK390808) and Papua New Guinea (MK061657, MK061654, AY714134), and the highest aa identity (100%) with *Haemoproteus* sequences from birds of Thailand (MK390808), South Korea (MW296829, MW296831, MW296830), and China (KT944102). Regarding *Leucocytozoon* spp., we obtained the same sequence type in three animals, more similar to the *Leucocytozoon* sp. sequence KU257627 (nucleotide and aa similarities of 97.1% and 95.6%, respectively) retrieved from a Spruce Grouse (*Falcipennis canadensis*) of Alaska, USA. The *Haemoproteus* phylogram showed that our Pyrenean Capercaillie sequence clustered together in well-supported clades with sequences of Western Capercaillie from Austria and with sequences of different species of raptors from Asia and Europe (Fig. 3B). The *Leucocytozoon* sp. sequence retrieved in Pyrenean Capercaillie clustered together, in well-supported clades, with *Leucocytozoon* sp. sequences obtained from different species within the family Phasianidae, from North America (Fig. 3C).

Representative herpesvirus, *Haemoproteus* sp. and *Leucocytozoon* sp. sequences obtained in this study were submitted to GenBank under accession numbers OR426924, OR354895 and OR354896, respectively.

## Discussion

To our knowledge, this is the first report of herpesvirus, *Haemoproteus* sp., and *Leucocytozoon* in Pyrenean Capercaillies. The detection of a novel herpesvirus sequence type in four animals from different counties and in a new host species (i.e., Pyrenean Capercaillie) supports its classification as a novel alphaherpesvirus species, tentatively named Tetrao urogallus alphaherpesvirus 1. Based on the phylogram and identity analysis results, this virus belongs to genus *Iltovirus*, which includes other species, such as *Gallid alphaherpesvirus 1*. *Gallid alphaherpesvirus 1* can cause infectious laryngotracheitis, a respiratory disease that affects chickens, pheasants, partridges, and peafowl^24^; however, the pathogenic potential of Tetrao urogallus alphaherpesvirus 1 remains unknown.

Avian malaria is caused by parasites of the genus *Plasmodium*, while avian malaria-like disease is caused by parasites of the genera *Haemoproteus* and *Leucocytozoon*; all of them comprised into the phylum Apicomplexa, order Haemosporidia^25^. In avian species, hemosporidian infections range from subclinical disease, anemia or slight plumage coloration change, to severe and even fatal disease^26,27^, potentially leading to severe declines in naïve wild bird populations^28^. Herein, we detected *Haemoproteus* in four Pyrenean Capercaillies. The genus *Haemoproteus*, a sister genus to malaria parasites (*Plasmodium*), parasitizes only birds and reptiles^29,30^. In birds, *Haemoproteus* species are considered cosmopolitan and are often prevalent^29,31^. *Haemoproteus* parasites are transmitted mainly by Culicoides biting midges (Ceratopogonidae), with only a few species vectored by louse flies (Hippoboscidae)^29,32^. This genus does not multiply in blood cells and presents predominantly light or moderate parasitemia^29^.

*Leucocytozoon* sp. was identified in three Pyrenean Capercaillies. This genus is transmitted by hematophagus black flies (Simuliidae)—with the exception of the species *Leucocytozoon caulleryi*, transmitted by biting midges (Culicoides)^29^. In the tribe Tetraonini, which comprises capercaillies, grouse and ptarmigan, the parasite *Leucocytozoon lovati* was detected in ptarmigan species inhabiting northern Norway and alpine areas of Japan^33,34^, while diverse *Leucocytozoon* haplotypes were identified in four grouse and three ptarmigan species of Alaska, USA^35^. This suggests that *Leucocytozoon* vectors are present in cold areas and at high altitudes similar to the Pyrenees.

Regarding the pathogenicity of the detected genera of hemoparasites, lesions associated with *Leucocytozoon* are more common than those of *Haemoproteus*^36^. High *Leucocytozoon* spp. parasitemia can cause emaciation, dehydration, convulsions, severe tissue damage, and mortality^36,37^. Additionally, a *Leucocytozoon* species—*Leucocytozoon lovati*—was associated with decreased reproductive success in the Black Grouse (*Lyrurus tetrix*)^38^, a galliform of the same family (Phasianidae) as the Pyrenean Capercaillie. The effects of *Leucocytozoon* sp. over the reproductive success of Pyrenean Capercaillies are still unknown. Although initially considered relatively benign to their hosts^39^, the *Haemoproteus* genus was recently associated with organ damage in naturally infected birds^10,40^. In our study, none of the *Leucocytozoon*- and/or *Haemoproteus*-positive animals analyzed by histopathology presented moderate or marked lesions associated with these parasites. These results are consistent with chronic parasitemia, a phase in which identifying persisting meronts in tissue samples can be difficult^41^. Nevertheless, potential costs to the immune-system or fitness of the animals should not be disregarded, especially in those individuals sustaining coinfection.

Although *Leucocytozoon* is better adapted to mountain conditions, the presence of insect-borne hemoparasites (*Haemoproteus* and *Leucocytozoon*) in the mountain galliforms analyzed herein was unexpected, particularly of *Haemoproteus*^42^. These animals were sampled in territories over 1570 m a.s.l., and one of the *Haemoproteus*-positive cases was sampled at 2255 m a.s.l. As explained above, these agents are mainly transmitted by the Culicoides biting midges and black flies, respectively^29,32^. The infection by the sister genus *Plasmodium*, transmitted by Culicidae and Ceratopogonidae^43^, was recently reported based on the hematology of a bearded vulture chick (*Gypaetus barbatus*) in the Pyrenees, also considered a mountain bird^44^. Culicoides biting midges are also important vectors of bluetongue virus, which causes an important disease of ungulates. In recent years, bluetongue virus has been detected in two of 89 Pyrenean chamois (*Rupicapra pyrenaica pyrenaica*) from the French Pyrenees, strongly suggesting the presence of the vector in the region^45^. It has been hypothesized that climate change may alter the geographic distribution of hemosporidians and increase parasite transmission (e.g., expanding vector ranges, promoting longer vector breeding seasons, and enhancing the development of avian haematozoa), consequently altering these parasites’ host range^3,46^. Worryingly, the average temperature in the Pyrenees has increased 1.2 °C from 1949 to 2010^47^, 30% over the average world increase (0.85 °C). Thus, long-term studies are warranted to address the impact of climate change on Pyrenean capercaillies and other mountain species inhabiting the Pyrenees, a hot spot of biodiversity.

All the Pyrenean Capercaillie fecal samples were PCR-negative to Avian influenza virus, Newcastle disease virus, *Ch. psittaci*, APEC, *Salmonella* spp. and *C. jejuni.* To the authors’ knowledge, there are no previous reports of the above mentioned agents in capercaillies. A previous study surveyed highly pathogenic avian influenza virus in 145 capercaillies in Europe, between 2016 and 2017, where all of the individuals tested negative to that agent^48^. Additionally, Avian influenza virus was not detected in the 79 mountain galliforms tested in the Italian Alps^49^. The negative results for APEC, *Salmonella* spp. and *C. jejuni* were not completely unexpected, once these pathogens have been previously described in other wild free-ranging galliforms, although generally at low prevalences^11,50–52^, and not necessarily considered part of their normal microbiota^51^. In capercaillies, the presence of *E. coli* was confirmed in captive and free-ranging birds of Germany, with higher occurrence in the captive individuals^50^. Nevertheless, the authors did not further characterize the isolates, and thus, it is not possible to know if these animals presented APEC. The same difference in *E. coli* prevalence between captive and free-raging birds was observed in red-legged partridges (*Alectoris rufa*) by Díaz-Sánchez et al.^51^, whom suggested that the bacterium could not be part of the normal intestinal microbiota of that species. In addition, the unique diet of Pyrenean Capercaillies, one of the few bird species able to eat coniferous needles^53^ is likely associated with a distinct intestinal microbiota, as observed in other capercaillie subspecies in Germany^53^. To this date, there is only one report of Western Capercaillie microbiome, which identified marked differences between cecal samples of captive and free-ranging birds from Germany^53^. Interestingly, the phylum Proteobacteria, which includes species like *E. coli*, *Campylobacter* spp. and *Salmonella* spp., only represented 4% of the microbiome of the analyzed free-ranging capercaillies, but 46% of the microbiome of the captive ones^53^. This could also be the case for Pyrenean capercaillies. Recently, García-Rodríguez et al.^11^ reported several cases of colibacillosis (caused by *E. coli*) in captive Cantabrian Capercaillies (i.e., isolated *E. coli* infections in nine chicks and one adult, and a co-infection by *E. coli*, *Clostridium perfringens*, and *Enterococcus gallinarum* in one chick). The potential impact of this bacterium in free-ranging Pyrenean capercaillie chicks is unknown. Further studies are necessary to characterize the Pyrenean Capercaillie intestinal microbiome. Additionally, larger sample sizes will be required to confirm our negative results.

Herein, we report the first description of herpesvirus, *Haemoproteus* and *Leucocytozoon* in Pyrenean Capercaillies, based on detailed molecular data and light microscopy. None of the tested fecal samples were positive to Newcastle disease virus, Avian influenza virus, *Ch. psittaci*, APEC, *C. jejuni*, and *Salmonella* spp. Our findings contribute to the current scarce knowledge about infectious agents in this species. In light of the endangered status of the Pyrenean Capercaillie and the expected consequences of climate change for hemosporidian parasite dynamics and mountain bird health, further studies are necessary to clarify the presence of clinical signs and pathologic changes associated with herpesvirus, *Haemoproteus* and *Leucocytozoon*. Although sedation methods have been developed to reduce the mortality risk related to capture myopathy and invasive sampling during Pyrenean Capercaillies captures^2^, this species is very sensitive to stress related to handling. Therefore, non-invasive methods and collection of carcasses should be prioritized to study Pyrenean Capercaillie diseases. Additionally, the presence of hemoparasites in mountain environments inhabited by Pyrenean capercaillies could also be assessed by sampling other birds species inhabiting the same areas (e.g., chicks in nests), and vectors. The surveillance results of these infectious agents will provide valuable insights for the development of in situ and ex situ management strategies, crucial for the conservation of this threatened galliform species.

### Supplementary Information

Supplementary Information.

## Data Availability

All data generated or analyzed during this study are included in this published article [and its supplementary information files]. The sequences obtained in the study were deposited in GenBank under accession numbers OR426924, OR354895 and OR354896.
