# Supplementary material for: F﻿irst detection ﻿of herpesvirus and hemosporidians in the endangered Pyrenean Capercaillie (Tetrao urogallus aquitanicus)
Source: Sci Rep. 2023 Dec 11;13:21936. doi: 10.1038/s41598-023-48123-3 (PMC10713636; doi:10.1038/s41598-023-48123-3)

SUPPLEMENTARY MATERIAL

Supplementary Table 1. Gross and histopathologic findings of the necropsied Pyrenean Capercaillies (*Tetrao urogallus aquitanicus*).

Case No.	Weight (kg)	Cause of death	Gross findings	Microscopic findings
22038 (La Tussa 2022)	3.4	Capture related death	Mild perioral hyperkeratotic proliferations, mainly at the base of the beak. Presence of some mucus at the choana. Small hyperkeratotic cutaneous sternal lesion. Minimum subcutaneous fat and a lack of intracelomic fat tissue.	Acute mild rhabdomyolysis with myofibrillar swelling, hyalinization, and fragmentation. Moderate vacuolar degeneration of interrenal cells and chromaffine cells hypertrophy. Marked multiorganic congestion. Mild sternal and perioral hyperplastic hyperkeratotic dermatitis.
22039 (Lles de Cerdanya 2022)	3.2	Capture related death	Stress bands in tail feathers. Small traumatic laceration at the tip of the tongue. Presence of some yellow mucus in the infundibulum and larynx. Moderate amount of subcutaneous and intracelomic fat tissue.	Acute mild cardiac and skeletal myolysis with myofibrillar swelling, fragmentation, and mitochondrial mineralization. Moderate vacuolar degeneration of interrenal cells. Marked multiorganic congestion.
22040 (Andorra 2021)	3.4	Traumatism (collision against a parked car)	Stress bands in tail feathers. Politraumatism with severe iIntracelomic and intracranial hemorrhages and multiple bone fractures.	Not performed.
22163 (Vall d'Aran 2020)	2.4	Emaciated, severe capillariosi	Cachexia, marked abdominal distension caused by abundant fluid contents in small intestine (severe capillariosis and presence of a few cestodes), mild acute fibrinous peritonitis.	Mild multifocal biliary trematodiasis, one parasitic granuloma in the lung (suspected larva migrans of Toxocara sp.*). Sections from the small and large intestines were too autolyzed for examination.
23001 (Drogo 2022)	Partial remains	Predation, most likely by a golden eagle	Emaciated. Hemorrhagic puncture dorsal thoracic lesions with severe lung hemorrhage. Bilateral deep inguinal lesions associated with the gps teflon	Extensive focal ulcerative bacterial and fungal post-traumatic dermatitis in inguinal skin, parasitic granuloma in the kidney (suspected larva migrans of Toxocara sp.*). Freezing

	(*Aquila chrysaetos*).	causing skin ulceration, hyperkeratosis, secondary infection, muscle degeneration, and bone periosteal remodeling of the left femur.	artifacts and autolytic changes difficult microscopic evaluation.

* The identification of the larva would require molecular techniques.

Supplementary Figure 1. Morphology of the Pyrenean capercaillie (*Tetrao urogallus aquitanicus*) feces at the field.

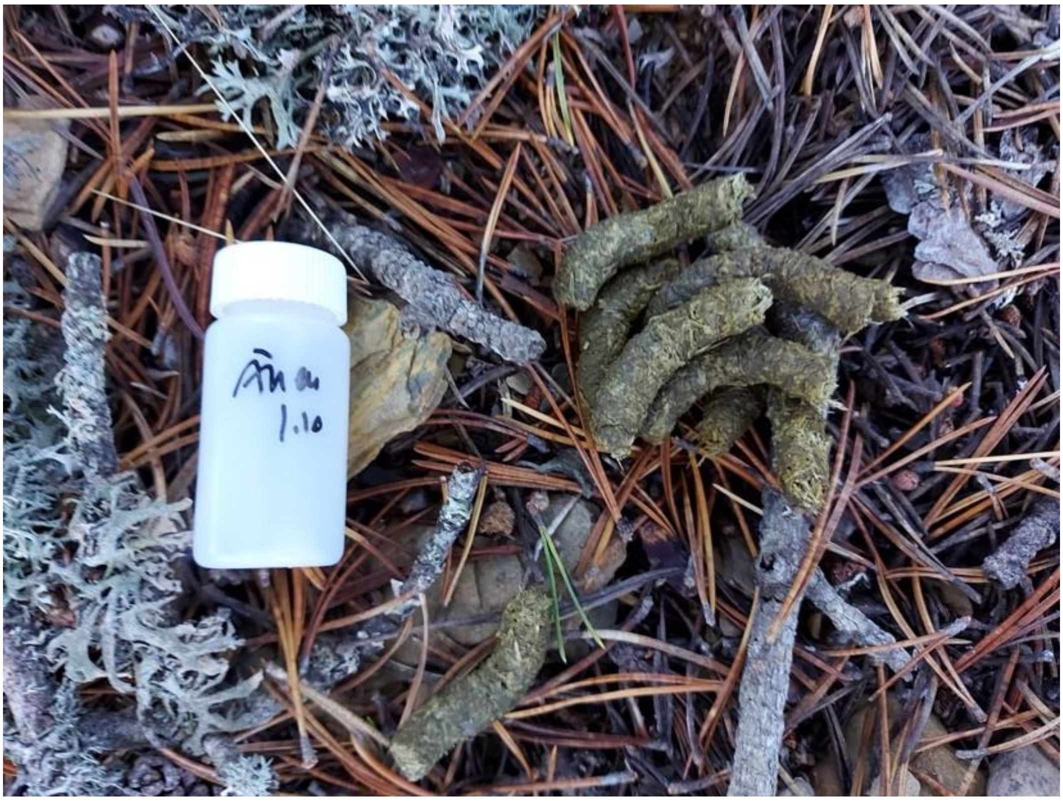

Supplement: Supplementary file 1 — Supplementary Information. [file 41598_2023_48123_MOESM1_ESM.pdf]
